# Making RF coils MR‐invisible by additive manufacturing using magnetically filled polymer

**DOI:** 10.1002/mrm.70115

**Published:** 2025-10-05

**Authors:** Markus Weiger, Johan Overweg, Amelie Viol, Lauro Singenberger, Thomas Schmid, Emily Louise Baadsvik, Klaas Paul Pruessmann

**Affiliations:** ^1^ Institute for Biomedical Engineering, ETH Zurich and University of Zurich Zurich Switzerland; ^2^ Uelzen Germany

**Keywords:** 3D printing, magnetite, PLA, short T_2_, UTE, ZTE

## Abstract

**Purpose:**

Short‐T_2_ MRI is sensitive not only to targeted tissues but also to signals from materials in RF coils, which can lead to image background artifacts. Current solutions to this problem either compromise imaging performance or impose restrictions on coil design. The goal of the present work is to make RF coils MR‐invisible without such drawbacks.

**Methods:**

Effective spoiling of unwanted signals from the housing of RF coils is achieved by filling the material used to construct the housing with magnetic particles. This concept is demonstrated by creating coil formers through additive manufacturing with custom filaments made from magnetite‐filled polymer.

**Results:**

Unwanted signals from the RF coil are effectively eliminated by using coil formers made from magnetically filled polymer. In this fashion, background‐free short‐T_2_ imaging is enabled.

**Conclusion:**

Making RF coils MR‐invisible by using magnetically filled materials simplifies coil design and manufacturing and renders the alteration of MR sequences unnecessary, thereby improving the performance of MRI of tissues with short T_2_s.

## 1 INTRODUCTION

In MRI, unwanted signals from parts of the MR scanner may be detected alongside signals from the targeted object. This concerns, in particular, the housing of RF coils, where signals get excited and/or received with high sensitivity by the nearby coil structure itself. Usually, the imaging FOV is selected to match the object size and therefore does not include the coil. As such, any signals detected from the coil will be aliased into the FOV, leading to artifacts of various patterns, depending on the type of sequence.^1^ For Cartesian k‐space encoding, this effect can be mitigated along the frequency‐encoding direction by bandpass filtering, but for the phase‐encoding direction, discrete fold‐over artifacts occur. For radial encoding, the appearance of the aliased signal is more diffuse, typically leading to an image background with possible variations in intensity and phase.^2^ A further aspect is blurring of coil signal, which depends on the signal lifetime and may extend into the imaged object as well as beyond the FOV.

Typically, RF coil housings are built from materials that exhibit relatively fast signal decay (T_2_s on the order of tens of microseconds), rendering them invisible at TEs above ≈ 1 ms. That said, there is a growing interest in MRI sequences with ultrashort or zero TE, which are employed to image tissues with very short T_2_ or T_2_* (on the order of tens to hundreds of microseconds), such as, for example, bone, tendon, ligaments, teeth, myelin, collagen, or lung.^3^ Therefore, artifacts caused by signals from RF coil housings and components have become a significant issue.^3^, ^4^, ^5^, ^6^ Because short‐T_2_ techniques typically use radial encoding, these artifacts mostly appear as a blurry image background.

There are several approaches to avoiding this problem: At the sequence level, they include compromising TE, using a large FOV,^3^, ^7^, ^8^ subtracting background signal,^2^ using T_2_‐selective magnetization preparation,^9^ or performing outer‐volume suppression.^10^ That said, these methods require undesirable sequence modifications, compromise sequence performance in terms of robustness and SNR efficiency, or offer only limited efficacy. Alternatively, materials with minimal content of the target nucleus can be used to construct RF coil housings, such as glass and polytetrafluorethylene for ^1^H imaging.^6^, ^11^, ^12^, ^13^, ^14^, ^15^ However, this approach imposes restrictions on coil design, manufacturing, robustness, cost, and handling, where the latter is particularly affected by the heavy weight of glass‐based coils.

In the present work, a new concept for eliminating background signals stemming from RF coils is proposed, relying on spoiling through local B_0_ distortion using magnetic perturbers. Such signal spoiling has previously been achieved with metallized textiles or by wrapping with magnetic wire.^13^ Here, this idea is pursued more fundamentally in that the material used for constructing the coil housing is filled with ferrimagnetic microparticles.^16^

The proposed concept is demonstrated by filling a polymer with magnetite and using it for additive manufacturing of RF coil formers.^17^, ^18^, ^19^ Formers with different base materials and filler load are assessed with respect to background signal, coil performance, and field homogeneity. The utility of the approach is demonstrated by imaging in phantom and in vivo.

## 2 METHODS

### 2.1 Test setup

**FIGURE 1:**
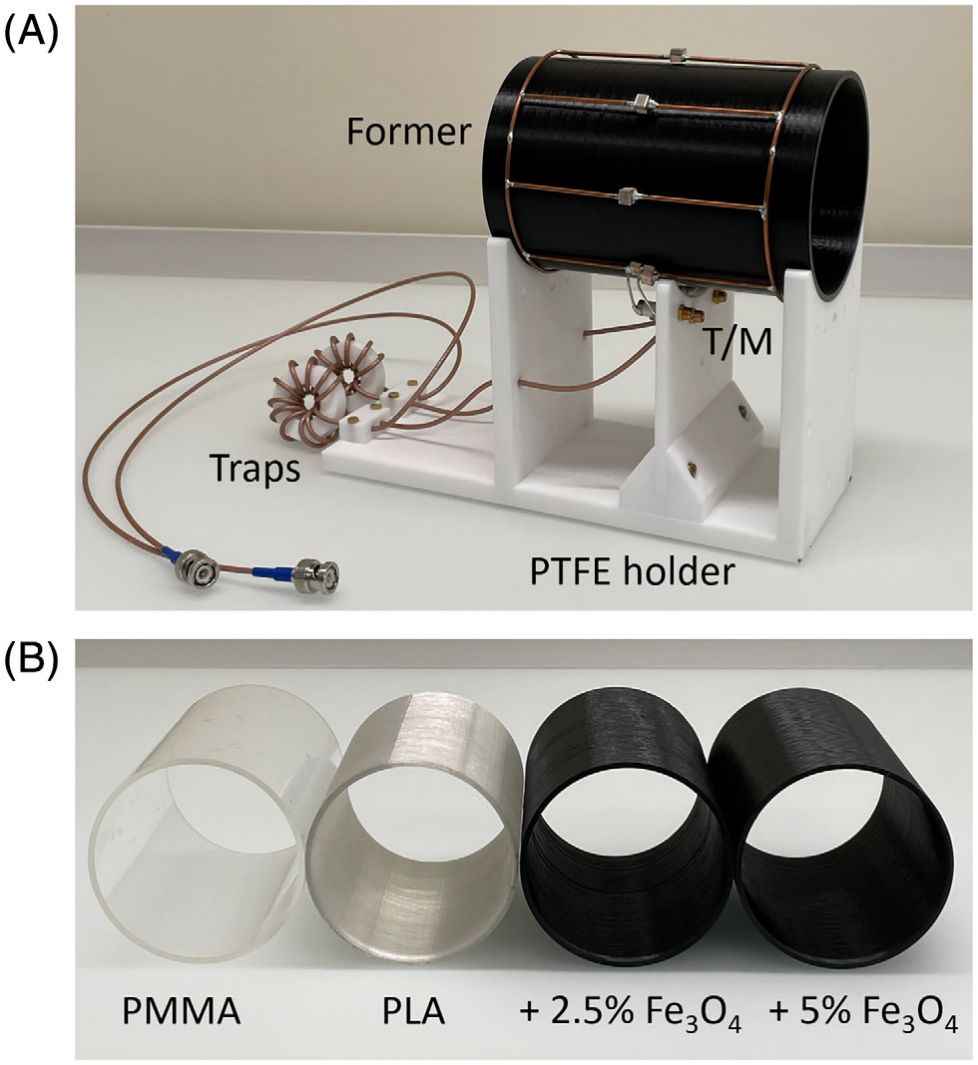
RF coil setup with exchangeable cylindrical former designed to assess different former materials. (A) Quadrature birdcage with low‐pass design including a former, a ^1^H‐free holder made from PTFE, a T/M circuit, and PTFE‐based cable traps. (B) Cylindrical formers made from the different materials assessed in this work. ^1^H, hydrogen‐1; Fe_3_O_4_, magnetite; PLA, polylactic acid; PMMA, polymethylmethacrylate; PTFE, polytetrafluorethylene; T/M, tuning and matching.

To demonstrate the proposed concept and compare formers made from different materials, a mechanically self‐supporting RF birdcage coil was designed, allowing for the exchange of a hollow cylinder that would normally act as the former (Figure 1A). Additional signals coming from the coil were minimized by using largely ^1^H–free materials in all other parts of the device, namely the holder, cables, cables traps, capacitors, and conductors.^13^ Electrically, the coil was designed as a quadrature, low‐pass birdcage with 102 mm diameter and 120 mm length. It was operated in a cylindrical RF shield of 330 mm diameter and 280 mm length made from conductive textile.^13^ A matching network that enables use with a large loading range, including the empty coil and a human wrist, was added.

### 2.2 Formers

**FIGURE 2:**
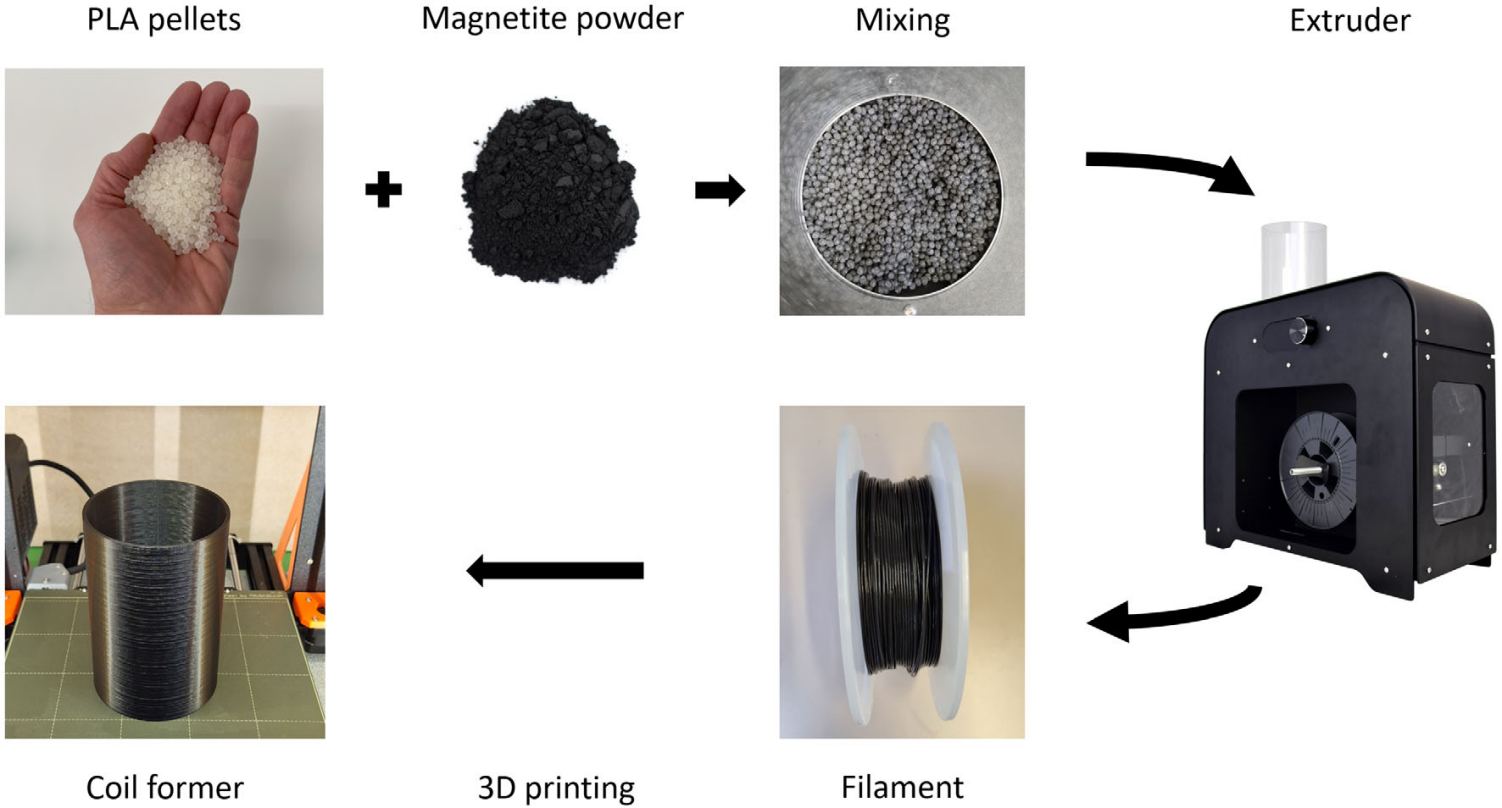
Procedure for manufacturing an RF coil former from magnetically filled polymer. Pellets made from PLA are mixed with magnetite powder and filled into an extruder (3devo, Utrecht, The Netherlands). The extruder produces a filament, which is then used to 3D print the hollow cylindrical former. Image sources: www.kremer‐pigmente.com, www.3devo.com. PLA, polylactic acid.

Four hollow cylindrical formers of 100 mm outer diameter, 3 mm thickness, and 150 mm length made from different materials were investigated (Figure 1B). Because polymethylmethacrylate (PMMA, (C_5_H_8_O_2_)n) is frequently used for making RF coils, this material served as a reference. The second former was obtained from polylactic acid (PLA, (C_3_H_4_O_2_)n) by additive manufacturing. For the custom‐made materials, PLA was filled with magnetite (Fe_3_O_4_) at either 2.5 or 5.0 % by volume by adding magnetite powder (particle size 1–20 μm; Kremer Pigmente, Aichstetten, Germany) to PLA pellets. The mixture was heated and extruded to produce filaments, which were then used to 3D print the hollow cylinders (Figure 2).

### 2.3 Experiments

MR experiments were performed using a 3 T Achieva system (Philips Healthcare, Best, The Netherlands) equipped with a high‐performance gradient,^20^ a custom RF chain,^7^ and fast transmit–receive switches.^21^ Two phantoms were used: a glass bottle of 65 mm diameter and 140 mm length filled with doped water, exhibiting a T_2_* ≈ 35 ms, and an object made from rubber (USB stick cap, ISMRM conference 2015, Toronto, Canada) of ≈ 70 mm diameter, exhibiting a T_2_* ≈ 500 μs. In vivo experiments were performed in a human wrist according to applicable ethics approval and with written informed consent from the subject. The tuning and matching of the coil were adjusted individually for each former and load. Free induction decay (FID) acquisition, short‐T_2_ imaging, and B_0_ mapping were performed using the parameters listed in Table 1. Furthermore, the unloaded and loaded quality factor, excitation efficiency, image SNR, and forces experienced in the magnet at the location of the strongest field gradient were determined: The excitation efficiency was obtained by finding the 90° flip angle in a pulse‐acquire experiment using a nonselective RF excitation and a long TR. The image SNR was calculated from magnitude images by taking the ratio of the average signal in a region of interest and the SD in a noise region of interest. The forces were derived from the angular deflection experienced by each former when suspended at the bore entry.^22^

**TABLE 1:** Experimental parameters. All imaging scans were performed with 3D isotropic geometry.

Protocol	FID	Large FOV	Matched FOV	In vivo	Field map
Figure	3	4	6	7	5
Sample	Coil formers	Water bottle	Rubber phantom	Human wrist	Water bottle
Sequence	Pulse‐acquire	PETRA	PETRA	PETRA	Gradient echo
Bandwidth [kHz]	4000	1000	250	500	172
FOV [mm]	–	210	80	130	160
Resolution [mm]	–	1.0	0.45	0.55	1.0
Dead time [μs]	6.3	10	10	10	–
TE [ms]	–	–	–	–	1, 2.38
Repetition time [ms]	1000	1	1	1	5
Flip angle [°]	7.5	3.6	2.9	3.3	5.0
Hard pulse duration [μs]	2	1	2	2	–
Angular undersampling	–	1.00	1.37	1.88	–
Number of signal averages	60	1	8	7	1
Scan time [m:s]	1:00	1:24	9:41	10:57	1:40

Abbreviations: FID, free induction decay; PETRA, pointwise encoding time reduction with radial acquisition.^31^

## 3 RESULTS

All quantitative results are summarized in Table 2. Overall, comparable coil performance with regard to quality factor and excitation efficiency was found for all formers. Only for PLA with the higher magnetite filling load was the excitation efficiency somewhat reduced. The minor impact of magnetite filling on RF performance is confirmed by the measured B_1+_ maps shown in Supporting Information: B_1_ Mapping. Magnetic forces on the magnetite‐filled formers are on the order of their own weight and hence nonnegligible.

**TABLE 2:** Experimental results obtained for different former materials when loading the coil with the doped water bottle (where applicable).

Material	PMMA	PLA	PLA 2.5%	PLA 5%
Q unloaded*	0.97	1.00	1.00	0.99
Q loaded*	1.00	1.00	1.00	1.00
Efficiency*	0.99	1.00	0.99	0.91
SNR*	0.94	1.00	0.97	0.95
FID amp. 6.3 μs*	1.46	1.00	0.190	0.014
FID amp. 10 μs*	1.33	1.00	0.060	0.011
FID T_2_* [μs]	11.2	14.3	2.8|3.8^†^	11.4|1.5^†^
B_0_ offset [kHz]	0	0	−1.4	−3.5
B_0_ SD*	0.98	1.00	3.18	7.93
Deflection angle [°]	0	0	35	65
Force/weight	0	0	0.7	2.1

*Note*: The symbol * indicates that the respective value is normalized relative to PLA. Absolute values for PLA were: Q unloaded = 68, Q loaded = 47, efficiency = 7.7 $\mathrm{\mu T}/\sqrt{W}$, SNR = 237, B_0_ SD = 49.4 Hz. The FID amplitudes were taken from the first data point acquired at 6.3 μs and at the imaging dead time of 10 μs. T_2_* values were determined by fitting the FIDs assuming exponential decay. Because this approach did not provide a meaningful result in case of PLA with higher filling, additional T_2_* values were derived from the signal loss at the first data point as compared to PLA, as indicated by the symbol †.

Abbreviations: PLA, polylactic acid; PMMA, polymethylmethacrylate; Q, quality factor.

**FIGURE 3:**
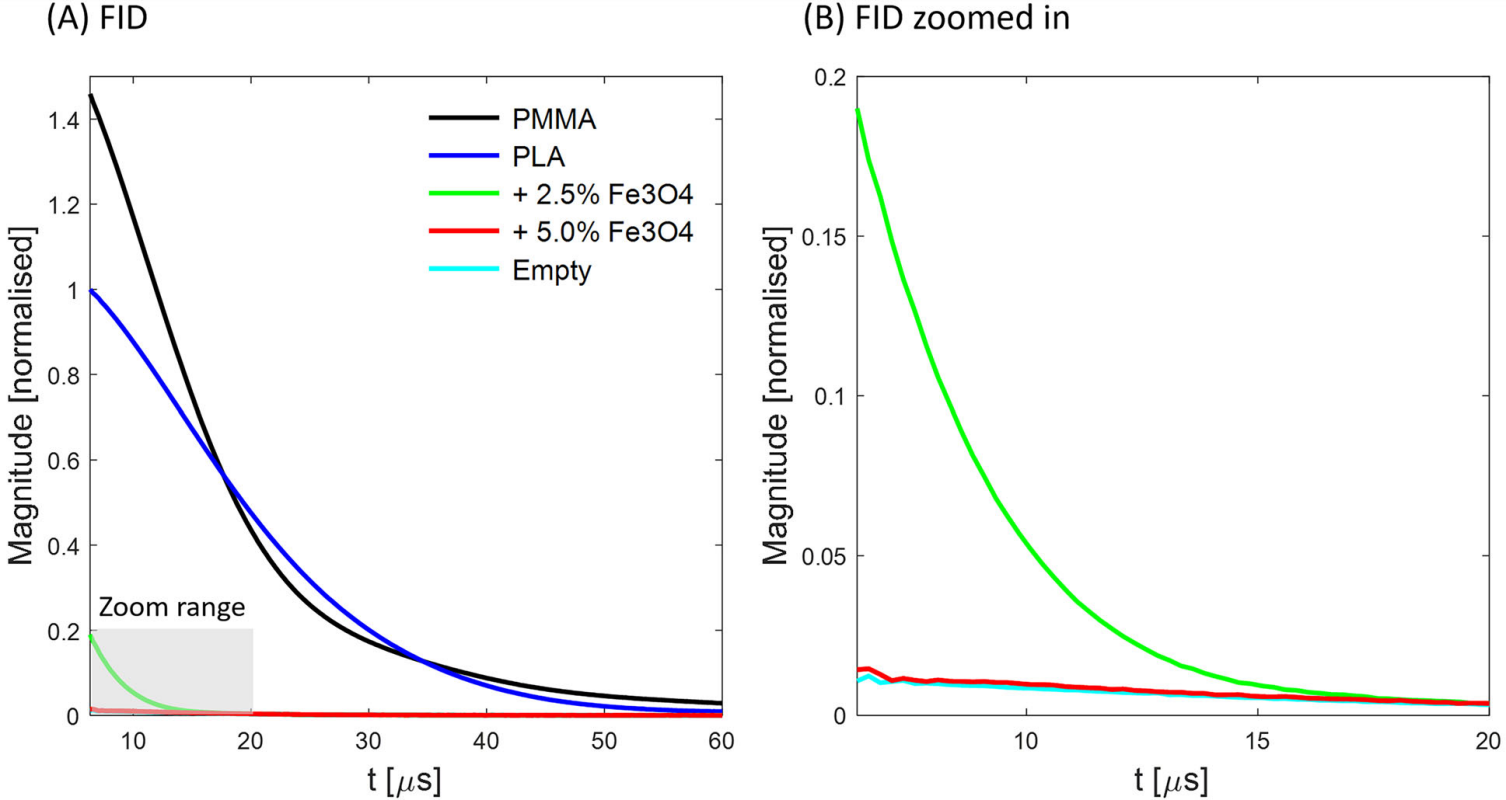
FID signals of different RF coil formers. The data was acquired with the RF coil shown in Figure 1 without an additional signal source. The coil was either entirely empty or had one of the formers inserted. Tuning and matching were adjusted individually for each load. The displayed signal magnitudes are normalized with the PLA maximum. (A) For the earliest acquired data point at 6.3 μs, PMMA has 46% higher signal than PLA. (B) The base PLA signal is strongly reduced for the magnetite‐filled PLA to 19% and 1.4% for the lower and higher filling factors, respectively. The signal level with higher filling approaches that of the empty coil. See Table 2 for further quantitative analysis. FID, free induction dccay.

Figure 3 shows FID signals of the formers without an additional load in the coil. PMMA exhibits higher signal than PLA, which is due to higher proton density. With magnetite filling, the initial signal amplitude of PLA is strongly reduced due to dephasing between excitation and the acquisition of the first data point (see also Table 2). With the higher filling load, the signal level drops close to that of the empty coil, which shows residual proton signal from the coil structure and holder. Estimated T_2_* values are reduced from ≈ 14 μs for pure PLA to ≈ 2–4 μs with magnetite filling. Correspondingly, the initial signal levels obtained with the filled materials occur only after several tens of μs for the unfilled references. Hence, imaging with TEs on this order using the unfilled materials would yield similar artifact suppression as the filling approach but would also reduce the targeted signal. The signal decays of the pure polymers are likely dominated by rapid intrinsic T_2_ relaxation, but probably also reflect reduced T_2_' due to macroscopic field distortion across the former. Comparing the shapes of the decay curves, the filled materials exhibit slight changes that indicate increase of both inhomogeneous and homogeneous broadening.

**FIGURE 4:**
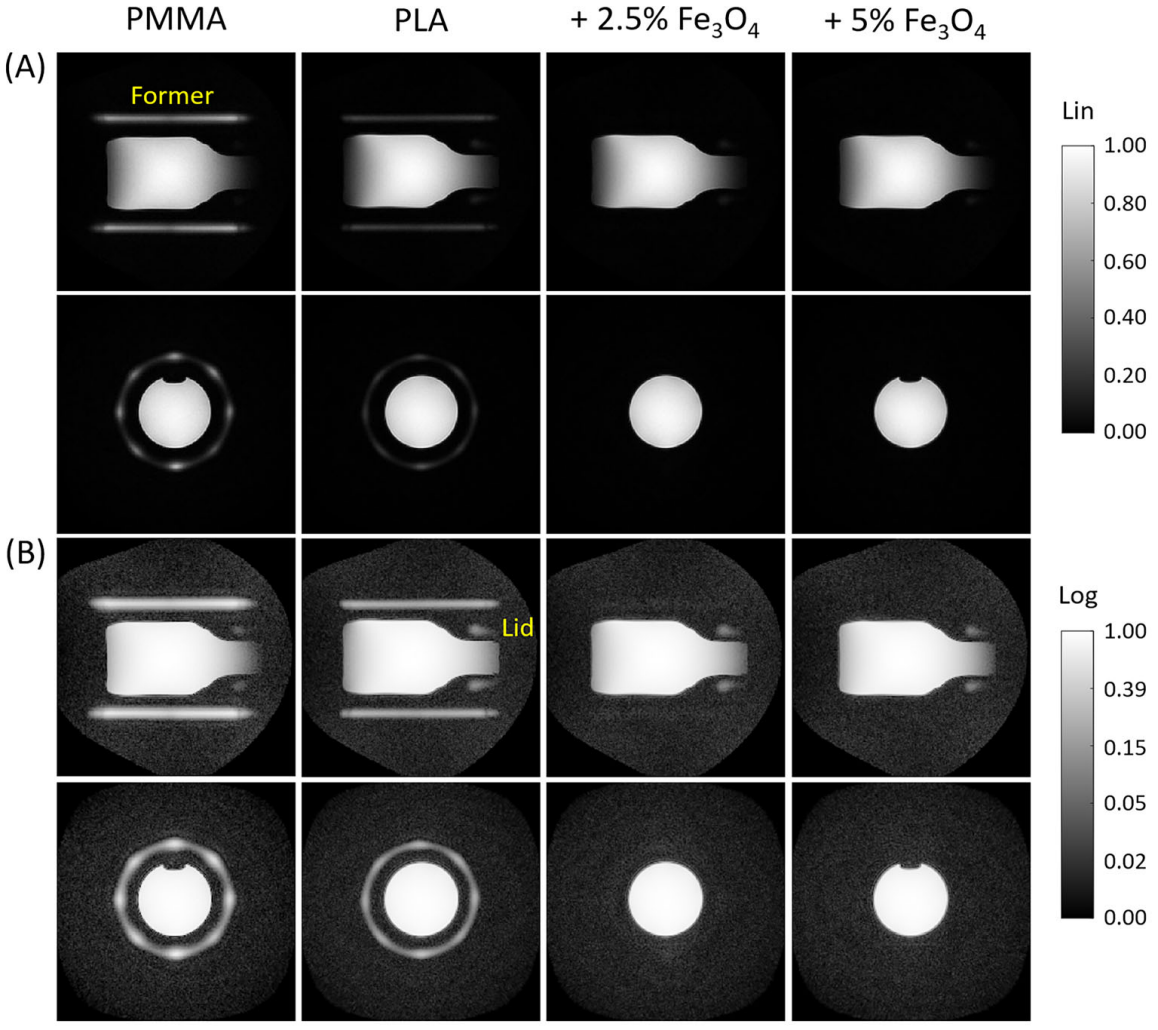
Image background of different RF coil formers in a large FOV. Short‐T_2_ imaging with a FOV fully covering the former was performed on a glass bottle, which was filled with doped water and closed with a plastic lid. For each former, a coronal and a transversal slice are shown with both (A) linear and (B) logarithmic gray scale to emphasize the image background. Both the PMMA and PLA formers are clearly visible, albeit with lower signal intensity for PLA. With 2.5% magnetite filling load, the PLA former signal is nearly removed but still faintly visible with logarithmic display. With 5% filling load, the former background is eliminated. Note that the air bubble in the bottle does not appear in all transversal images due to repositioning when exchanging the formers.

In the short‐T_2_ images with large FOV shown in Figure 4, the pure PMMA and PLA formers are clearly visible. For the PLA formers with magnetite filling, the signal drops to noise level for the lower filling load and vanishes entirely for the higher filling load. These findings match the FID results of Figure 3 and the amplitudes at the imaging dead time as provided in Table 2.

**FIGURE 5:**
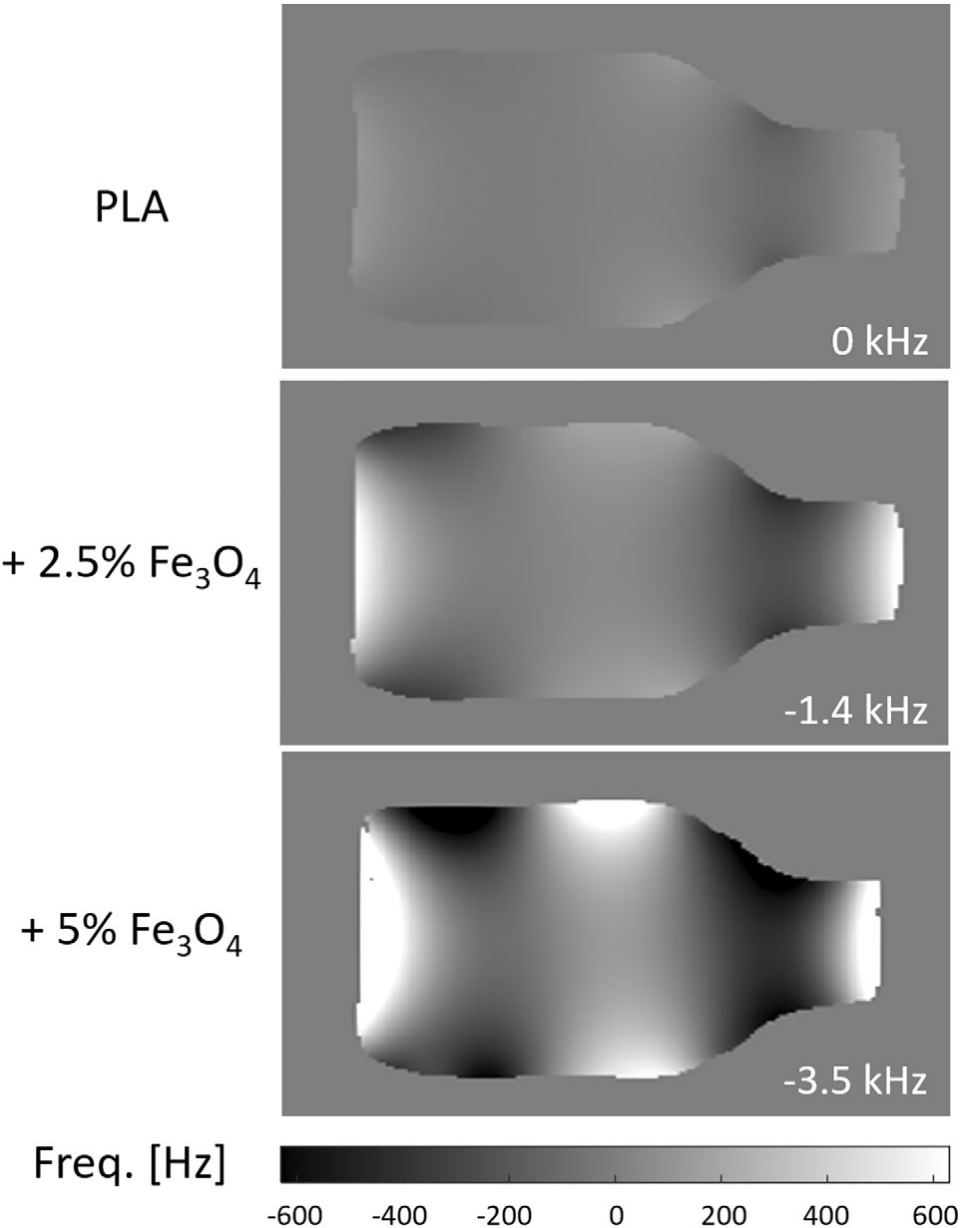
Field maps acquired in the water bottle with the three PLA formers after second‐order shimming. Pure PLA shows slight field deviations caused by the cylindrical former and the bottle. With magnetite filling, the nonuniformity is increased but keeps the same pattern. Furthermore, a bulk field offset occurs (value on the bottom right). Both effects are increased with higher filling load (see also Table 2). Note that the values in the masked bottle area do not reflect the given offsets.

Figure 5 shows the effects of the magnetic material on the static field in the imaging volume after second‐order shimming, which increase with higher filling load (see also Table 2). Two main effects are observed: a constant field offset, which can readily be accounted for by adjusting the scanner frequency, and field distortions of hundreds of Hz. The resulting field inhomogeneity is larger than one would usually target in standard MRI but is less critical at the typically higher gradient strengths used in short‐T_2_ imaging. Potential image artifacts induced by increased B_0_ inhomogeneity are illustrated in Supporting Information: B_0_ Artifacts.

**FIGURE 6:**
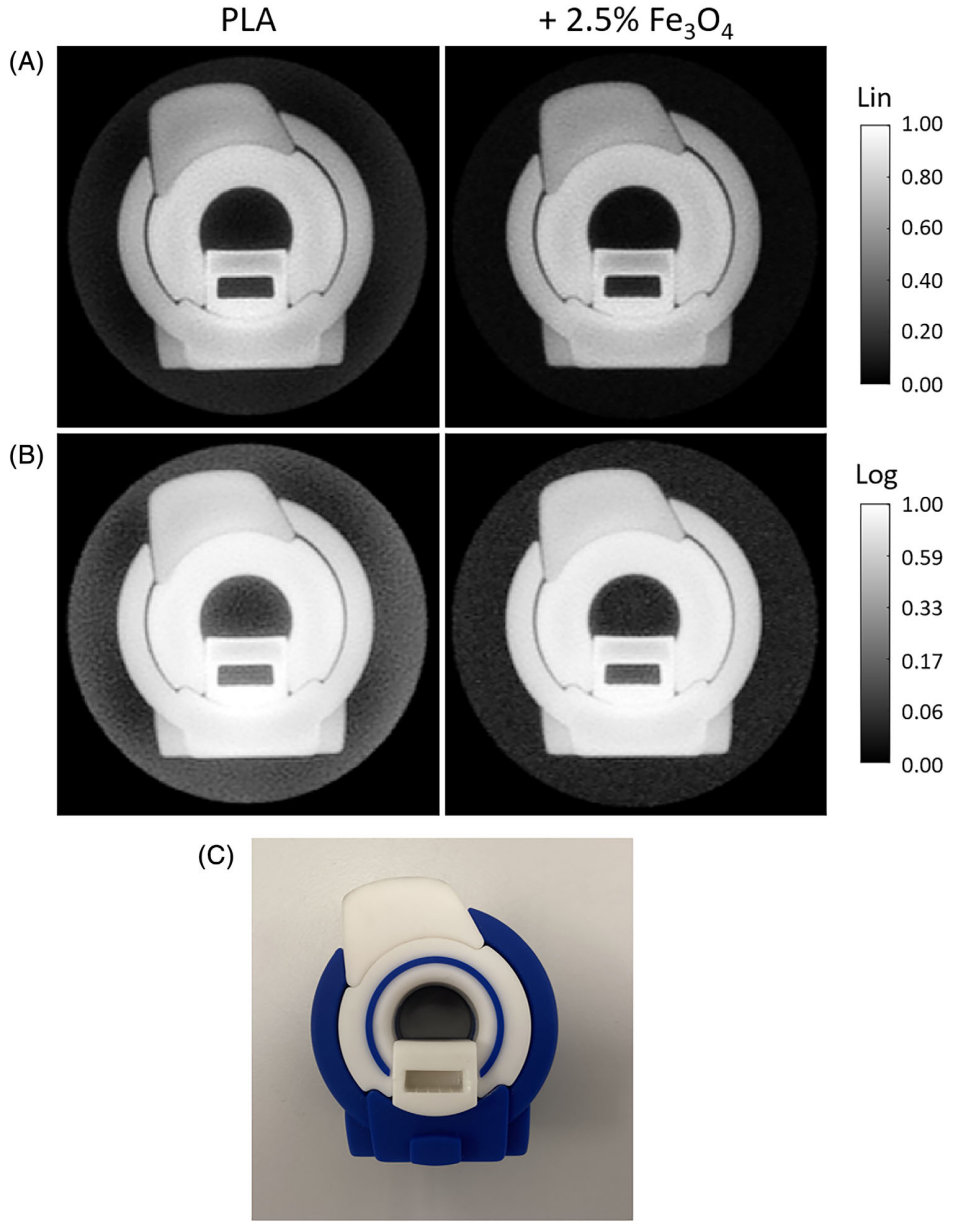
Image background from RF coil formers in an object‐matched FOV. Short‐T_2_ imaging was performed in a rubber phantom using PLA formers without and with magnetite filling. The FOV was chosen to cover the phantom size and thus does not enclose the former. The images are displayed with (A) linear and (B) logarithmic gray scale to emphasize the image background. With pure PLA, the signal of the former is aliased into the FOV, leading to background artifacts in the entire FOV. With magnetite filling, the PLA signal is significantly reduced, providing a clean background. (C) Photograph of the phantom.

**FIGURE 7:**
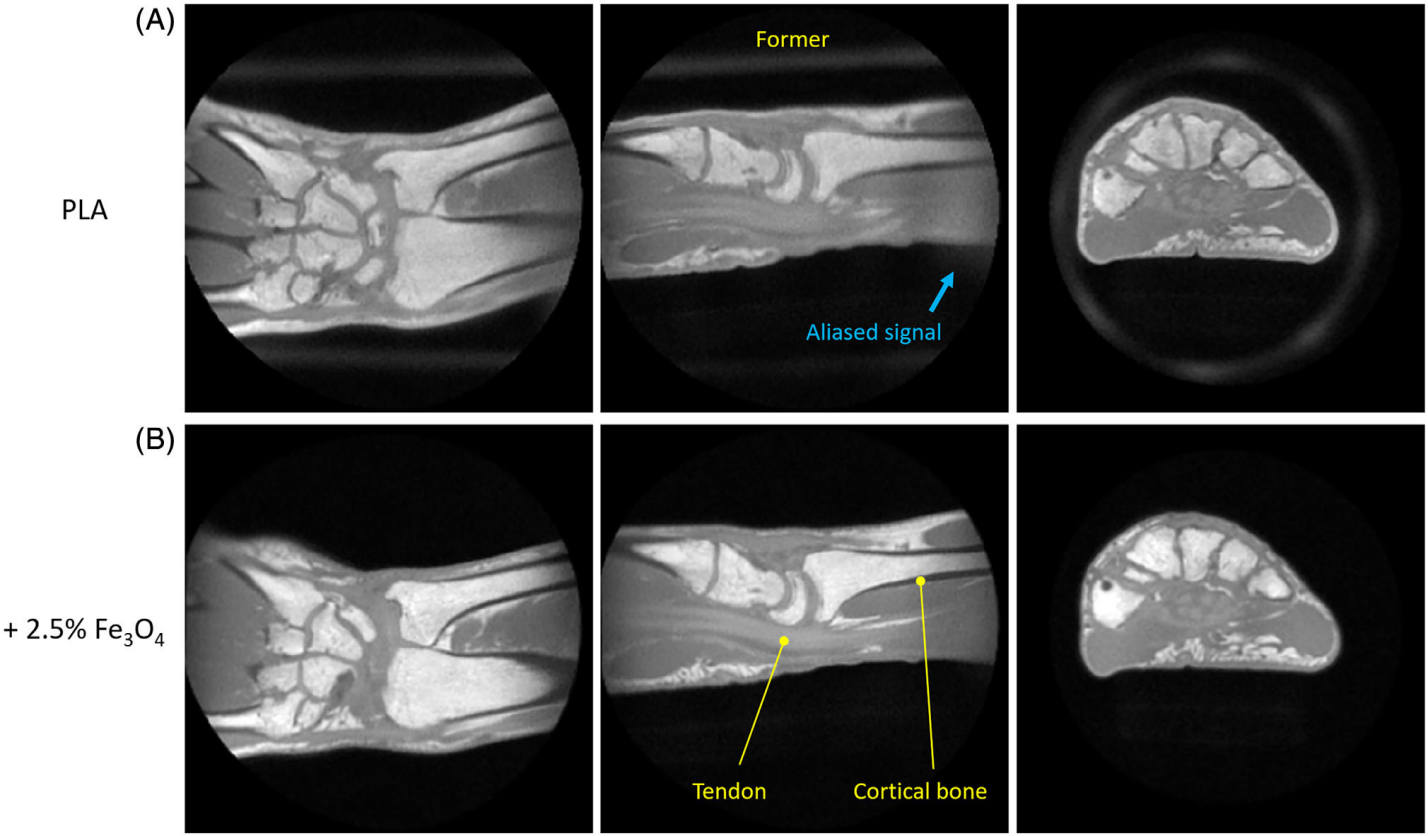
In vivo short‐T_2_ imaging of a human wrist using different formers. The FOV was chosen to approximately cover the sensitivity range of the birdcage coil along the axial dimension. Three different orientations extracted from the 3D data sets are displayed. (A) In addition to the wrist, the PLA former is also visible, which is partly covered by the FOV. Furthermore, aliased signal is observed both outside and inside the object. (B) With the PLA former with 2.5% magnetite filling, no signal from the coil is visible, and a clean background is obtained. Short‐T_2_ tissues such as tendon and cortical bone are depicted. Note that the two data sets were acquired in different sessions, limiting the reproducibility of the object positioning and possibly leading to different motion effects.

Given the results of Figures 4 and 5, the material with 2.5 % filling load is considered a good compromise between signal reduction and field distortion and was therefore used for the successive imaging experiments. Figure 6 shows that for short‐T_2_ imaging with object‐matched FOV, signal from the PLA coil former is aliased into the FOV and appears as a background artifact. This artifact is successfully eliminated when using the magnetite‐filled former. The experiment presented in Figure 7 demonstrates the advantage of using this coil former for in vivo imaging, showing clean short‐T_2_ images unaffected by background signal.

## 4 DISCUSSION

In this work, a new concept for making RF coils MR‐invisible is proposed and successfully demonstrated for short‐T_2_ imaging with short dead time and high bandwidth.

The concept relies on the field emanating from magnetic particles causing strong spatial field variations in their neighborhood on the microscopic level. Across an observed volume, this leads to a loss of coherence between spins and, hence, rapid decay of signal on the macroscopic level, which can be approximately characterized by very short T_2_*. This effect is fundamentally the same as achieved with superparamagnetic contrast agents.^23^ The relative contributions of homogeneous and inhomogeneous broadening to the shorter T_2_* depend on particle dimensions and distribution as well as physicochemical properties of the surrounding material. For the desired spoiling effect, this distinction is less relevant. To optimize spoiling, the roles of the underlying mechanisms could be considered more closely, for example, by suitable simulations. As demonstrated in the present work, T_2_* can be shortened such as to render MR signal from coil material negligible even for acquisitions with dead times on the order of microseconds. In the case of predominantly inhomogeneous broadening, refocusing by successive RF pulses could be a concern. That said, for common plastics, T_2_s are short relative to typical TRs even before filling, thereby excluding spurious echoes. Notably, significant T_1_ reduction is not expected from magnetic filling due to long correlation times in the solid‐state material, except at much lower B_0_.

Compared to spoiling by surrounding magnetic material,^13^ the key advancement in this work is manipulating the bulk material itself so that the desired effect is reliably obtained everywhere in the manufactured part regardless of geometry. Naturally, other properties potentially altered by magnetic filling should also be considered, namely mechanical robustness, thermal behavior, permittivity, and electrical resistance, with the latter two potentially affecting gradient eddy currents and/or RF losses. In the present work, no obvious changes related to these properties were observed apart from slightly increased surface roughness and minor changes in coil performance.

As for the choice of magnetic material, magnetite turned out to be particularly suitable for the targeted purpose. It has a high saturation magnetization of ≈ 600 mT^24^ while offering superior behavior concerning corrosion, flammability, and conductivity compared to iron. Saturation of magnetization occurs at an external field of ≈ 400 mT^25^; thus, the spoiling efficiency of a given filled material will be comparable across common clinical field strengths. At saturation, the concept of susceptibility as a means of quantifying the magnetic properties of the filled materials has limited validity. Alternatively, saturation magnetizations of approximately 15 mT and 30 mT can be derived by weighted averaging using the magnetite concentrations used in the present work, which are about five orders of magnitude larger than for the base materials.^26^, ^27^

Magnetically filled parts could also be manufactured in other ways such as, for example, by casting. However, producing filled polymer filaments for additive manufacturing offers high flexibility in coil design and minimal production effort. Notably, this approach could be used to manipulate material properties also for other purposes in MR, for example, to improve field homogeneity by tuning the magnetic susceptibility of in‐bore parts along with their geometry.^28^

For the present application of targeting short T_2_*, magnetic filling has the side effect of impairing the homogeneity of the main field within the imaging volume (see Figure 5). The field pattern is a result of the shape of the magnetized parts (here, the former; more generally, all filled parts of the coil housing), whereas the strength of the distortion scales with their magnetization. An infinite cylinder along the main field axis would not affect uniformity.^29^ Thus, field homogeneity would be improved already by increasing the length of the former within given geometric constraints. Further improvements could be achieved by profiling the former's thickness along its axis and/or locally varying the magnetite concentration. For a full housing, an outer former, covers, and internal support structures will need to be taken into account. Other coil geometries and orientations with respect to the main field will create different field distortions. All these aspects should be considered during coil design, preferably supported by magnetostatics simulations. Relatively simple geometries such as a hollow cylinder allow for analytical modeling,^30^ whereas more complicated structures require numerical calculations. An example of such a calculation is illustrated in Supporting Information: B_0_ Simulation.

Apart from controlling the shape of filled parts to contain field inhomogeneity, it would be an option to reduce their magnetization. In the present example, the filling load was chosen such as to balance signal suppression and field distortion. Beyond this, the amount of magnetic material could be reduced by minimizing the wall thickness or by printing at lower density, that is, including small‐scale voids. Furthermore, filling requirements could be reduced by use of polymers with lower proton density, such as, for example, polycyclohexylendimethylen terephthalate glycol. In this context, it would again be useful to investigate the impact of the size of the magnetic particles on spoiling efficiency.

Imaging at high quality is possible despite significant field inhomogeneities introduced by filled parts, as demonstrated in Figures 4, 5, 6, 7. For robust imaging, readout gradients must be strong relative to the field distortion. The pixel bandwidth of all imaging protocols in Table 1 is > = 1 kHz, and they therefore fulfill this requirement as indicated in the field map for the lower filling load in Figure 5. Although gradients are often weaker in standard MRI, the rapid encoding needed for short‐T_2_ imaging inevitably entails the use of strong gradients, thus rendering local resonance offsets less critical. On the other hand, contrast in short‐T_2_ MRI is sometimes created through magnetization preparation by means of RF pulses, which may be susceptible to B_0_ inhomogeneity. In this case, the more robust, adiabatic implementations of such pulses can be a solution. Another way of generating contrast is based on images with different TEs, which would be undesirably affected by a T_2_* reduced by the coil housing. Collectively, these aspects speak for exploiting the discussed means of minimizing the amount of magnetic material.

A set of issues that need thorough consideration regarding handling and safety are the forces and torques experienced by a magnetized housing. The related measurements (see Table 2) showed that when moving the formers into the bore of an actively shielded 3 T magnet, maximum forces occurred that were on the order of the formers' weight. Although the associated pull can be readily handled by the operator, an uncontrolled movement may happen when being inattentive. Notably, the forces scale with the external field, rendering operation at high field strengths more critical. As such, appropriate mounting provisions and installation procedures will be necessary to guarantee safety for both hardware and subjects. In any case, a reduction of the magnetic material as elaborated above would relax the situation.

With the removal of the coil signal, other sources of background signal such as padding, scanner components, or parts of the body extending beyond the coil may account for the largest remaining image contamination. For foam materials, a similar magnetite filling‐based approach might be envisaged. Signals from the scanner could be suppressed by RF shielding. Finally, unwanted signals from the body could be minimized by optimization of the sensitivity range of the coil.

## 5 CONCLUSION

A new concept was proposed for making RF coils MR‐invisible that does not require alteration of MR sequences and greatly simplifies coil design and manufacturing as compared to previous approaches. The associated reduction of field homogeneity in the imaging volume currently favors application for dedicated short‐T_2_ imaging. However, possible improvements concerning the amount and distribution of the magnetic material could enable usage for any type of imaging sequence, thereby rendering magnetic filling more universally applicable in RF coil design.

## Supporting information


**Figure S1.** B1^+^ maps acquired with the test setup using formers made from different materials. The same central coronal slice is displayed for each material. The central region is largely uniform and a typical drop‐off towards the ends of the coil is observed. With all materials, a similar overall field pattern is obtained. Only the map obtained with the larger magnetite filling exhibits some ripples and additional non‐uniformity at the bottom of the bottle, which is due to artifacts in the base images induced by the higher B0 inhomogeneity (see Figure 5).
**Figure S2.** 3D gradient echo images acquired with the frequency encoding direction applied as indicated. The central coronal slice is displayed with logarithmic scaling to better show the ends of the bottle, where B1 is relatively low. Two kinds of artifacts are observed, which both increase with the increased inhomogeneity associated with higher magnetite filling as well as with reduced pixel bandwidth (PBW). First, image distortions occur in particular at the bottleneck (blue arrow). Second, signal loss due to local dephasing appears at the bottom of the bottle (yellow arrow). The observed effects are in correspondence with the B0 field maps shown in Figure 5.
**Figure S3.** Numerical simulation of the field inhomogeneity generated by a hollow cylinder (the coil former) with a uniform magnetisation of 15 mT. (A) Central x‐z plane indicating the former position (on one side only), the phantom range of the related experiments, and the locations of the field profiles drawn in (B).
