# Supplementary material for: Making RF coils MR‐invisible by additive manufacturing using magnetically filled polymer
Source: Magn Reson Med. 2025 Oct 5;95(2):1279–88. doi: 10.1002/mrm.70115 (PMC12681291; doi:10.1002/mrm.70115)
Supplement: Supplementary file 1 — Figure S1. B1+ maps acquired with the test setup using formers made from different materials. The same central coronal slice is displayed for each material. The central region is largely uniform and a typical drop‐off towards the ends of the coil is observed. With all materials, a similar overall field pattern is obtained. Only the map obtained with the larger magnetite filling exhibits some ripples and additional non‐uniformity at the bottom of the bottle, which is due to artifacts in the base images induced by the higher B0 inhomogeneity (see Figure 5). Figure S2. 3D gradient echo images acquired with the frequency encoding direction applied as indicated. The central coronal slice is displayed with logarithmic scaling to better show the ends of the bottle, where B1 is relatively low. Two kinds of artifacts are observed, which both increase with the increased inhomogeneity associated with higher magnetite filling as well as with reduced pixel bandwidth (PBW). First, image distortions occur in particular at the bottleneck (blue arrow). Second, signal loss due to local dephasing appears at the bottom of the bottle (yellow arrow). The observed effects are in correspondence with the B0 field maps shown in Figure 5. Figure S3. Numerical simulation of the field inhomogeneity generated by a hollow cylinder (the coil former) with a uniform magnetisation of 15 mT. (A) Central x‐z plane indicating the former position (on one side only), the phantom range of the related experiments, and the locations of the field profiles drawn in (B). [file MRM-95-1279-s001.pdf]

Supplementary Information for

Making RF coils MR-invisible by additive manufacturing using magnetically filled polymer

Markus Weiger[1], Johan Overweg, Amelie Viol[1], Lauro Singenberger[1], Thomas Schmid[1],

Emily Louise Baadsvik[1], Klaas P. Pruessmann[1]

[1]Institute for Biomedical Engineering, ETH Zurich and University of Zurich, Zurich, Switzerland

B1 MAPPING

To investigate the influence of the magnetic former on the RF field pattern, B1^{+} mapping was performed. The same bottle as described in the 'Experiments' section was used, but filled with doped water with $T_2^* \approx 350$ ms. The actual flip angle technique (1) was employed using an RF-spoiled 3D gradient echo sequence with TE = 1.3 ms, TR = 30 and 150 ms, nominal flip angle $\alpha = 60°$, FOV = $100\times96\times160$ mm^3, resolution = $2.5\times2.0\times1.2$ mm^3, bandwidth 300 kHz, and scan time 7 m 4 s.

The resulting maps shown in Figure S1 demonstrate that the B1^{+} field is only slightly affected by the surrounding former material, which is in agreement with the finding concerning RF efficiency (see Table 2).

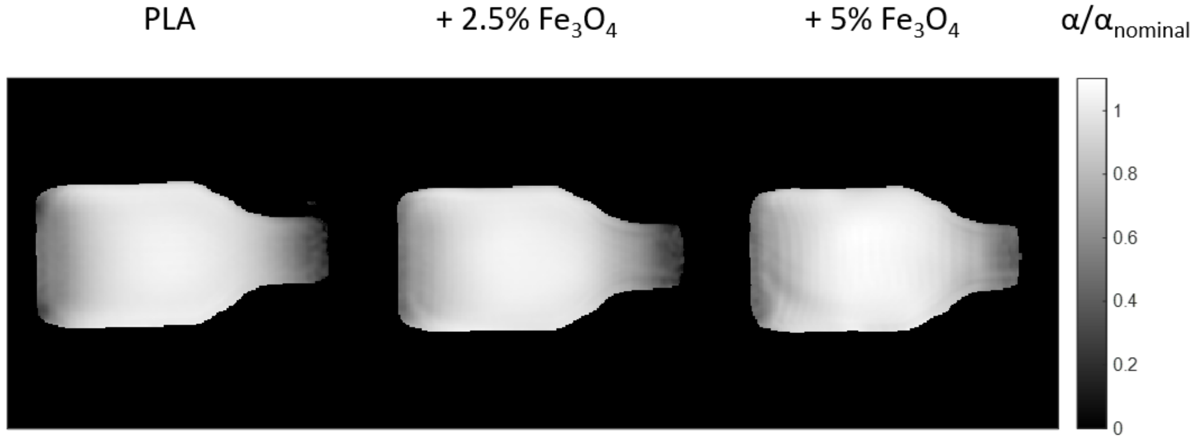

Figure S1: B1^{+} maps acquired with the test setup using formers made from different materials. The same central coronal slice is displayed for each material. The central region is largely uniform and a typical drop-off towards the ends of the coil is observed. With all materials, a similar overall field pattern is obtained. Only the map obtained with the larger magnetite filling exhibits some ripples and additional non-uniformity at the bottom of the bottle, which is due to artefacts in the base images induced by the higher B0 inhomogeneity (see Figure 5).

B0 ARTEFACTS

To illustrate potential artefacts induced by increased static field inhomogeneity arising from magnetite filling, 3D gradient-spoiled gradient echo images were acquired from the phantom bottle using the three PLA formers. The same protocol as for B0 field mapping was used (see Table 1) but with only one echo at TE = 2.2 ms. The experiments were performed at different strengths of the frequency encoding gradient of 25.1 and 6.4 mT/m, which led to values for the pixel bandwidth (PBW) of 1072 and 272 Hz, respectively.

Figure S2 shows images with two kinds of artefacts with their strength depending on PBW and the degree of magnetite filling, namely image distortions and signal loss. No artefacts are observed at large PBW for PLA without and with lower filling. Note that at low PBW, slight image distortions are observed even without filling.

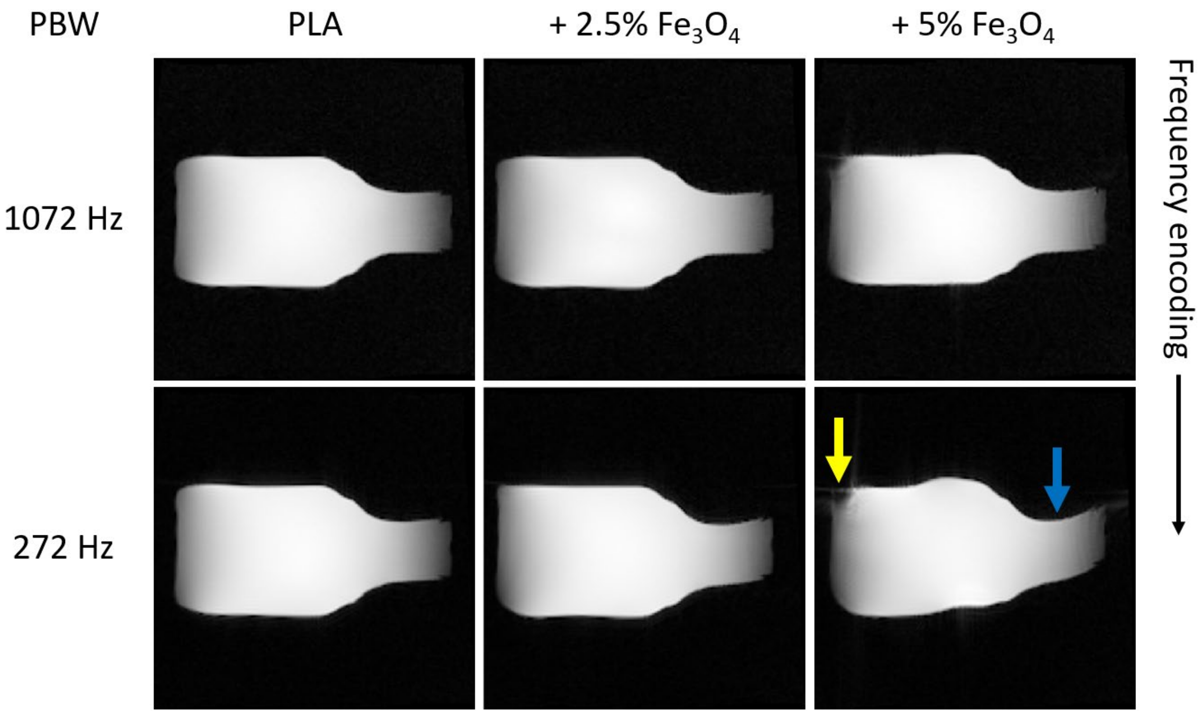

Figure S2: 3D gradient echo images acquired with the frequency encoding direction applied as indicated. The central coronal slice is displayed with logarithmic scaling to better show the ends of the bottle, where B1 is relatively low. Two kinds of artefacts are observed, which both increase with the increased inhomogeneity associated with higher magnetite filling as well as with reduced pixel bandwidth (PBW). First, image distortions occur in particular at the bottleneck (blue arrow). Second, signal loss due to local dephasing appears at the bottom of the bottle (yellow arrow). The observed effects are in correspondence with the B0 field maps shown in Figure 5.

B0 SIMULATIONS

To illustrate prediction of expected B0 inhomogeneity, numerical simulations of magnetostatics were performed. A hollow cylinder was used with the former geometry as described for the test setup and a magnetisation of 15 mT, corresponding to filling polymer with 2.5vol% of magnetite.

Figure S3 shows the resulting field distribution with the largest gradients in proximity to the former and better homogeneity towards the centre, where the imaging volume is located. The obtained characteristics match the experimental results in Figure 5.

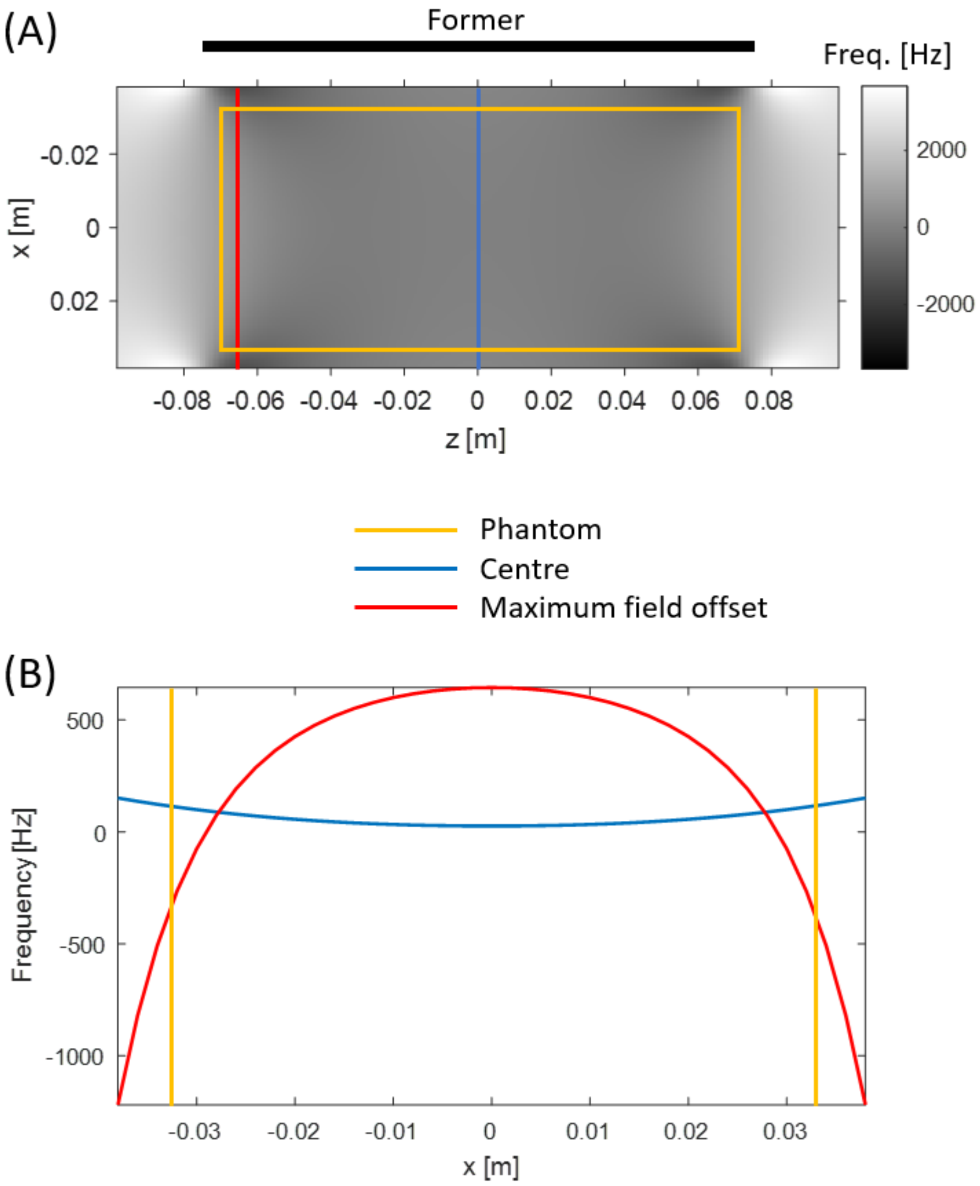

Figure S3: Numerical simulation of the field inhomogeneity generated by a hollow cylinder (the coil former) with a uniform magnetisation of 15 mT. (A) Central x-z plane indicating the former position (on one side only), the phantom range of the related experiments, and the locations of the field profiles drawn in (B).

REFERENCES

1. Yarnykh VL. Actual flip-angle imaging in the pulsed steady state: A method for rapid three-dimensional mapping of the transmitted radiofrequency field. Magn Reson Med 2007;57:192-200.
